# *Escherichia coli* and *Staphylococcus aureus* Differentially Regulate Nrf2 Pathway in Bovine Mammary Epithelial Cells: Relation to Distinct Innate Immune Response

**DOI:** 10.3390/cells10123426

**Published:** 2021-12-06

**Authors:** Yi-Tian Ying, Jing Yang, Xun Tan, Rui Liu, Ying Zhuang, Jia-Xue Xu, Wei-Jia Ren

**Affiliations:** 1 Department of Veterinary Medicine, Zhejiang University, Yuhangtang Road 866, Hangzhou 310058, China; yingyitian@zju.edu.cn (Y.-T.Y.); 21917098@zju.edu.cn (J.Y.); 3170100083@zju.edu.cn (R.L.); 22017117@zju.edu.cn (Y.Z.); 22117114@zju.edu.cn (J.-X.X.); 22017103@zju.edu.cn (W.-J.R.); 2 Veterinary Medical Center, Zhejiang University, Yuhangtang Road 866, Hangzhou 310058, China; 3 Institute of Preventive Veterinary Sciences, Yuhangtang Road 866, Hangzhou 310058, China; 4 Hainan Institute of Zhejiang University, Yazhou Bay Science and Technology City, Sanya 572025, China

**Keywords:** bovine, mastitis, mammary epithelia cells, Nrf2, *Escherichia coli*, *Staphylococcus aureus*

## Abstract

*Escherichia coli* and *Staphylococcus aureus* are major mastitis causing pathogens in dairy cattle but elicit distinct immune and an inflammatory response in the udder. However, the host determinants responsible for this difference remains largely unknown. Our initial studies focused on the global transcriptomic response of primary bovine mammary epithelial cells (pbMECs) to heat-killed *E. coli* and *S. aureus*. RNA-sequencing transcriptome analysis demonstrates a significant difference in expression profiles induced by *E. coli* compared with *S. aureus*. A major differential response was the activation of innate immune response by *E. coli*, but not by *S. aureus*. Interestingly, *E. coli* stimulation increased transcript abundance of several genes downstream of Nrf2 (nuclear factor erythroid 2-related factor 2) that were enriched in gene sets with a focus on metabolism and immune system. However, none of these genes was dysregulated by *S. aureus*. Western blot analysis confirms that *S. aureus* impairs Nrf2 activation as compared to *E. coli*. Using Nrf2-knockdown cells we demonstrate that Nrf2 is necessary for bpMECs to mount an effective innate defensive response. In support of this notion, nuclear Nrf2 overexpression augmented *S. aureus*-stimulated inflammatory response. We also show that, unlike *E. coli*, *S. aureus* disrupts the non-canonical p62/SQSTM1-Keap1 pathway responsible for Nrf2 activation through inhibiting p62/SQSTM1 phosphorylation at S349. Collectively, our findings provide important insights into the contribution of the Nrf2 pathway to the pathogen-species specific immune response in bovine mammary epithelial cells and raise a possibility that impairment of Nrf2 activation contributes to, at least in part, the weak inflammatory response in *S. aureus* mastitis.

## 1. Introduction

Mastitis (inflammation of the mammary gland and udder tissue) is the most prevalent disease in dairy cows and is a major cause of economic losses in dairy farms worldwide [1]. A wide spectrum of pathogenic agents has been implicated in the etiology of mastitis, of which *Escherichia coli* and *Staphylococcus aureus* are the most commonly involved gram-negative and gram-positive bacteria, respectively [2,3,4,5]. It is well known that these two pathogens elicit a distinct inflammatory response in the bovine udder. *E. coli* infection leads very often to severe inflammation associated with rapid onset of clinical signs followed by fast clearance of the infection [6], although some *E. coli* strains have been reported to cause chronic infection [7]. On the contrary, intramammary infections with *S. aureus* typically cause a much weaker innate immune response, resulting in lifelong pathogen persistence with severe tissue damage [8,9]. Infected cows routinely shed large numbers of *S. aureus* into their milk, which can lead to foodborne poisoning in humans [10], posing a threat to human health. Moreover, cows have been identified as the main source of novel human-pathogenic *S. aureus* clones [11], underscoring their zoonotic potential. Although extensively studied, the interaction between *S. aureus* and the host response remains largely unclear.

The nuclear factor erythroid 2-related factor 2 (Nrf2, encoded by *Nfe2l2*) is a stress responsive transcription factor, which is well known for its function of cytoprotection against electrophilic and oxidative stress [12]. Nrf2 activity is suppressed under homeostatic conditions through Keap1 (Kelch-like erythroid cell-derived protein with CNC homology-associated protein 1)-mediated ubiquitination-proteasomal degradation. In the presence of electrophiles or oxidants, Nrf2 disassociates from Keap1, resulting in Nrf2 stabilization and nuclear translocation/accumulation, followed by transcriptional activation of a battery of Nrf2 target genes encoding detoxifying enzymes and antioxidant proteins, such as NAD(P)H:quinone oxidoreductase (NQO)-1, glutamate-cysteine ligase, modifier subunit (GCLM) and superoxide dismutase (SOD) [13,14]. Besides mediating a stress-stimulated induction of antioxidant and detoxification genes, Nrf2 is recognized as a master regulator of tissue damage control to infection [15]. In addition, it has been shown that Nrf2 activation limits an excessive inflammatory response [16,17]. However, there is also evidence showing that Nrf2 promotes, rather than restrains, the immune response [18]. So far, little is known about the Nrf2 pathways in the pathophysiology of bovine mastitis.

Bovine mammary epithelial cells (MECs) lining the inner surface of the mammary gland constitute an important part of innate immunity. MECs are the most abundant cell type of the lactating udder [19]. These cells are capable of responding to bacterial intrusion, and are regarded as active contributors to immune and inflammatory responses of the mammary gland [20,21,22,23]. Furthermore, recent evidence shows the pathogen-specific immune response of MECs, but not of the resident immune cells (e.g., macrophages), which reflects many aspects of the pathogen species-specific characteristics of in vivo infected udders [20,24], leading to the suggestion that the respective response of MECs determines the pathogen species-specific immune response of mastitis [25].

In this study, we compared the transcriptome profile of primary cultures of bovine MECs (pbMECs) stimulated with heat-killed mastitis pathogens *E. coli* and *S. aureus*. We found that pbMECs responded to *E. coli*, but not to *S. aureus*, with transcriptional activation of several Nrf2 target genes. We further explored the mechanisms by which *E. coli* cause Nrf2 activation, whereas *S. aureus* do not, by focusing on the well characterized canonical redox-Keap1-Nrf2 [12,26] and non-canonical p62/sequestosome 1 (SQSTM1)-Keap1-Nrf2 pathways [27]. In addition, the role of Nrf2 in the immune response of bovine mammary epithelium to mastitis pathogens was investigated.

## 2. Materials and Methods

### 2.1. Cell Culture and Mastitis Pathogens

pbMECs were isolated from 3 healthy first lactating Chinese Holstein heifers using procedures as previously described [28]. The procedure was approved by the Animal Care and Use Committee guidelines in Zhejiang University (approval number ZJU20160379). The cows had been culled in the normal culling regime. Cells were cultured in low-glucose Dulbecco’s modified Eagle’s medium (DMEM) containing 10% fetal calf serum (FCS), insulin (1 μg/mL), amphotericin B (10 μg/mL, Invitrogen, Carlsbad, CA, USA), tylosin tartrate (50 μg/mL) and penicillin–streptomycin (200 μg/mL) at 37 °C in a humidified atmosphere of 5% CO_2_. After 2 h, DMEM was replaced by fresh medium and the medium was then changed every 2 days. The primary cell cultures were passaged at ~80% confluence. Fibroblasts in the culture were removed as previously described [29]. The pure pbMECs after passages 4 were used for subsequent experiments. The *E. coli* and *S. aureus* used in this work were isolated from the milk sample of bovine mastitis. Identification of bacterial species was performed by means of culture, Gram staining and 16S rRNA gene sequencing (data not shown). The culturing of the bacteria and their use to challenge pbMECs were conducted as described previously [30]. *E. coli* and *S. aureus* inactivation was performed at 70 °C and 85 °C, respectively, for 30 min. Viability loss of pathogens after heat treatment was tested on blood agar plates.

### 2.2. High throughout Sequencing

pbMECs were stimulated with heat-inactivated *E. coli* or *S. aureus* at 1 × 10^7^ particles/mL for 24 h [30]. Total RNA was extracted using TRIzol reagent (Takara Biomedical Technology, Dalian, China) following the manufacturer’s protocol without modification. Sequencing library of each sample was constructed with 1 μg total RNA using NEBNext UltraTM RNA Library Prep Kit (NEB, llumina Inc., San Diego, CA, USA). The proper length of cDNA in each library was ensured by running the DNA 1000 assay on the Agilent 2100 Bioanalyzer. The library preparations were sequenced on an Illumina Hiseq Xten platform and 150 bp paired-end reads were generated. After quality assessment of the raw reads using FastQC (version 0.10.1, Babraham Institute Bioinformatics Group, Cambridge, UK), adapter sequences and sequences of low quality (Sanger base quality < 20) were trimmed. The clean reads of each sample were then mapped to the Bos Taurus reference genome (assembly ARS-UCD1.2) using TopHat2 (version 2.0.9, Johns Hopkins University Center for Computational Biology, Baltimore, MD, USA) with default parameters. Only reads with a perfect match or one mismatch were further analyzed and annotated. Analysis of differential expression genes (DEGs) between groups was conducted using the DESeq R package (version 1.10.1, Bioconductor, Boston, MA, USA). The resulting *p* values were adjusted using the Benjamini and Hochberg’s approach to control the false discovery rate (FDR). The genes with absolute Log2 fold change (log2FC) > 0.1 and FDR < 0.05 were considered as significant DEGs between groups. The DEGs was then subjected Gene ontology (GO) analysis, Kyoto Encyclopedia of Genes and Genomes (KEGG) pathway and Reactome pathway enrichment analysis.

### 2.3. Real-Time Quantitative PCR

Total RNA was extracted using TRIzol reagent (Takara Biomedical Technology, Dalian, China). First strand cDNA synthesis was performed using PrimeScript RT reagent Kit with genomic DNA Eraser (Takara Biomedical Technology, Dalian, China) in a reaction containing 1 μg RNA according to the supplier’s instruction. Intron-spanning primer sets were designed using the Primer-BLAST program (http://www.ncbi.nlm.nih.gov/tools/primer-blast, 20 March 2019) and are presented in Table S1. Primer amplification efficiencies were determined by real-time quantitative PCR (qPCR) using the equation E = 10^[−1/slope]^-1. PCR was performed using Roche LightCycler 480 with SYBR Green Realtime PCR Master Mix Plus (Vazyme, Nanjing, China). Data were normalized to reference genes *RPL19* and *PGK1* by using an efficiency corrected method of Pfaffl [31].

### 2.4. Immunoblot Analysis

Cells were washed twice with phosphate-buffered saline (PBS) and lysed in radioimmunoprecipitation assay buffer (RIPA) (FDbio Science, Hangzhou, China) containing protease inhibitors and phosphatase inhibitors. Samples were boiled at 95 °C for 5 min to denature proteins. Proteins were separated by using a sodium dodecyl sulfate (SDS)-10% polyacrylamide gel electrophoresis (PAGE) Fast Preparation Kit (FDbio Science, Hangzhou, China) and electroblotted onto polyvinylidene difluoride (PVDF) membranes (Millipore, Bedford, MA, USA). After being blocked for 2 h in Tris-buffered saline and Tween 20 (TBST) containing 5% non-fat milk, membranes were washed three times in TBST and probed with primary antibodies against Keap1 (Proteintech, Wuhan, China), Nrf2 (R1312-8, HUABIO, Hangzhou, China), pIκBα (Santa Cruz Biotechnology, Shanghai, China), phospho-NF-κB p65 (Ser536) (Abcam, Cambridge, UK), GADPH (FDbio Science, Hangzhou, China), Histone (Santa Cruz Biotechnology, Shanghai, China), SQSTM1/p62 (Affinity Biosciences, Changzhou, China), pSer349-SQSTM1/p62 (Affinity Biosciences, Changzhou, China), GFP (HUABIO, Hangzhou, China) and β-actin (Cell Signaling Technology, Danvers, MA, USA). The primary antibody was detected using appropriate horseradish peroxidase (HRP)-conjugated secondary antibody. Immunoreactive bands were visualized by electrochemiluminescent (ECL) (FDbio Science, Hangzhou, China).

### 2.5. Nuclear and Cytosolic Fractionation

Cells were washed 3 times with PBS before nuclear and cytosolic fractionation. Nuclear and cytoplasmic fractionation were conducted using the Nuclear-Cytosol Extraction Kit (FDbio Science, Hangzhou, China) according to the manufacturer’s instructions. Each separated protein was analyzed by Western blot analysis.

### 2.6. Immunofluorescent Staining

pbMECs were seeded on 24-well plates at 1 × 10^4^ cells/well. After treatments, the cells were fixed with 4% paraformaldehyde followed by permeabilization in 0.25% Triton X-100 in PBS. After incubated with 5% bovine serum albumin (BSA), the cells were probed with a primary antibody against Nrf2 (1:1000, HUABIO, Hangzhou, China) overnight at 4 °C. Then the cells were incubated with an Alexa Fluor 594-conjugated secondary antibody (1:1000, Abcam, Cambridge, UK). Cells nuclei was visualized with 4,6-diamidino-2-phenyindole (DAPI, Sigma-Aldrich, St. Louis, MO, USA). Cytosolic and nuclear Nrf2 location was examined using a fluorescence microscope (Nikon Eclipse Ti-S, Tokyo, Japan).

### 2.7. Measurement of ROS Levels

ROS was measured using 2′,7′-dichlorofluorescein diacetate (DCF-DA, Sigma-Aldrich, Sigma-Aldrich, St. Louis, MO, USA). Briefly, pbMECs were incubated with LPS (10 μg/mL) or *S. aureus* (1 × 10^7^ particles/mL) for 6–12 h. After washing in PBS, the cells were incubated in 20 μM DCF-DA in serum-free medium for 15 min in the dark at 37 °C. 2,7-dichlorofluorescein (DCF) fluorescence was measured at 480 nm/520 nm by flow cytometry (BD, FACS Verse, San Jose, CA, USA).

### 2.8. Introduction of Small Interfering RNA against Nrf2

Small interfering RNAs (siRNAs) were designed and synthesized by GenePharma (Shanghai, China). Cells were transfected with target-specific or nontargeting negative control siRNAs using Lipofectamine 2000 (Invitrogen, Waltham, MA, USA) according to the manufacturer’s instructions. The sequence of Nrf2 siRNA is 5′-GCAAUUCAACGAGGCUCAATT-3′ (sense) and 5′-UUGAGCCUCGUUGAAUUGCTT-3′ (antisense). The sequence of negative control siRNA is 5’- UUCUCCGAACGUGUCACGUTT -3’ (sense) and 5’-ACGUGACACGUUCGGAGAATT-3’ (antisense). The final concentration of the siRNAs was 20 nmol/L. Knockdown efficiencies were determined by qPCR and Western blot analysis.

### 2.9. Determination of Cell Viability

Cell viability was determined using the methyl thiazolyl tetrazolium (MTT) assay. Briefly, cells were incubated with 10 mg/mL MTT (Sigma-Aldrich, Saint Louis, MO, USA) at 37 °C for 4 h. Dimethyl sulfoxide (DMSO) was added to culture medium to dissolve the formazan crystals for 15 min in the dark. The optical density (OD) of the solubilized product was measured at 540 nm. Cell viability was plotted as the mean OD540 value of six replicates.

### 2.10. Plasmids Construct and Transient Transfection

The full-length open reading frame (ORF) of bovine p62/SQSTM1 cDNAs with an optimal Kozak consensus sequence just before the in-frame first ATG was cloned into the eukaryotic expression vectors pCMV-N-Myc. For the construct of 3× NLS-Nrf2 expression plasmid, a DNA fragment encoding 3 repeat (3×) nuclear localization signal (NLS) polypeptide PKKKRKV from the SV 40 large T antigen was subcloned into a *XhoI*/*HindIII* digested pEGFP-C3 plasmid, followed by inserting the Nrf2 ORF cDNA into the *HindIII*/*BamHI* digested pEGFP-NLS plasmid. The constructs were confirmed by DNA sequencing. Expression plasmid DNAs were transfected into pbMECs using the Lipo8000 (Beyotime Biotech, Nanjing, China) according to the manufacturer’s instructions. Cells were used for further analysis at 24 or 48 h after transfection.

### 2.11. Statistical Analysis

Data are presented as mean ± standard deviation (s.d.). Statistical analysis was performed with the SPSS software version 22 (IBM Corp, Armonk, NY, USA). The significance of the differences between groups was analyzed using a one-way ANOVA followed by LSD, or a nonparametric Mann–Whitney *U* test, as appropriate. A value of *p* < 0.05 was taken as the threshold level for statistical significance.

## 3. Results

### 3.1. Gene Expression Profiles in E. coli- and S. aureus-Challenged pbMECs

**Figure 1:**
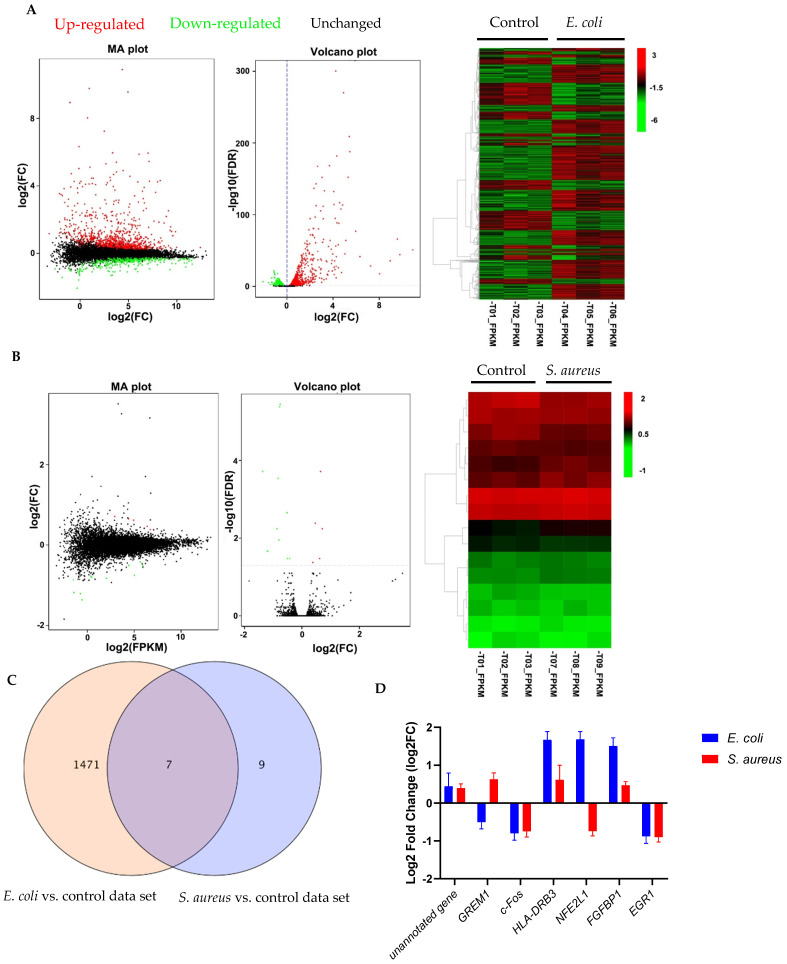
Gene expression profiling analysis of differential expression genes. (**A**,**B**) MA plots, volcano plots and hierarchical clustering showing differential expression profiles in primary cultures of bovine mammary epithelial cells (pbMECs) stimulated with heated-killed *E. coli* (**A**) or *S. aureus* (**B**) compared to unstimulated cells. Differentially expressed genes are established at log2(FC) > 0.1 and false discovery rate (FDR) < 0.05. FC, fold change; FPKM: Fragments Per Kilobase Million. (**C**) Venn diagram comparing the gene expression changes induced by heated-killed *E. coli* or *S. aureus*. (**D**) Expression patterns of the overlapping differentially expressed transcripts between *E. coli*- and *S. aureus*-stimulated cells compared with control showing in (**C**). Results are expressed as log2FC over unstimulated control cells. Data are mean ± s.d. of triplicates.

To characterize the modulatory effect of *E. coli* and *S. aureus* on the gene expression profiles, pbMECs were stimulated with heat-killed *E. coli* or *S. aureus* for 24 h. Unstimulated cells were served as controls. *E. coli* particle treatment induced a large number of gene expression changes. We identified 1478 (8.5% of 17297 genes) differentially expressed genes (DEGs) from the transcriptome profiles of cells stimulated by heat-inactivated *E. coli* compared to unstimulated cells (Figure 1A), comprising 933 up- (Additional file 1) and 545 downregulated (Additional file 2) genes. In contrast, stimulation of heat-inactivated *S. aureus* particles induced few gene expression changes as compared to control cells. Actually, only 16 genes exhibited altered expression at 24 h after *S. aureus* challenge (Figure 1B), of which 5 had not been annotated in the reference cow genome. Among the remaining DEGs, 4 were up-regulated and 7 down-regulated (Table S2). The Venn diagram in Figure 1C illustrates the large number of DEGs induced by *E. coli*. Of these, only 0.48% overlapped with *S. aureus*-induced expression changes, suggesting that *E. coli*-induced expression profiles were significantly different from those of *S. aureus*. The overlapping transcripts are shown in Figure 1D.

### 3.2. Functional Analysis of Differentially Expressed Genes Induced by E. coli

**Figure 2:**
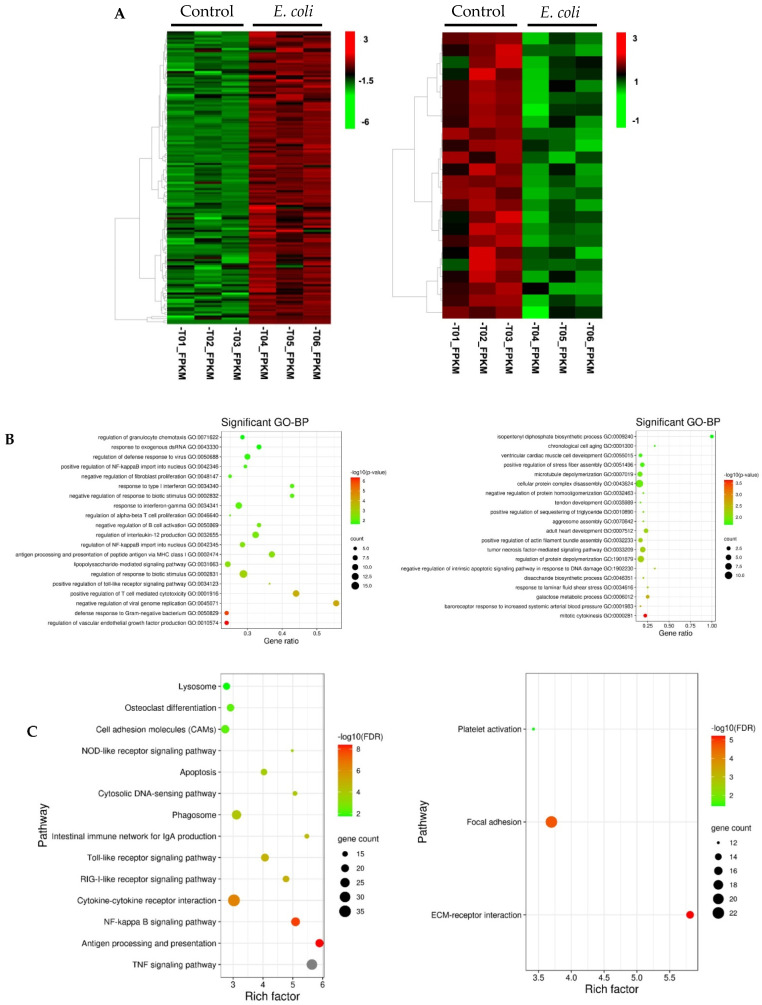
Function annotation and pathway analysis for the up- and downregulated genes between normal vs. *E. coli* samples. (**A**) Hierarchical clustering of gene expression for the up- (left panel) and downregulated (right panel) genes. Row represent individual genes and columns represent the expression changes of replicate for each investigated group. Red color indicates relative over-expression, while green color indicates relative under-expression. (**B**) Bubbleplot for GO enrichment of the upregulated (left panel) the downregulated genes (right panel) with statistically significant biological processes (FDR < 0.05). The top 20 GO enrichment terms are presented. y-axis: ontological terms; x-axis, the gene ratio of enriched among the background genes in each ontological term. (**C**) Bubbleplot for KEGG pathway enrichment of the up- (left panel) and downregulated (right panel). Significant enrichment of a pathway was defined as FDR < 0.05. y-axis, functional pathways; x-axis, rich factor. The size of the bubble is proportional to the number of genes assigned to the GO/KEGG entry and the color corresponds to the adjusted *p* value. A high FDR is represented by red, and a low value is represented by green. GO, Gene Ontology; KEGG, Kyoto Encyclopedia of Genes and Genomes.

Hierarchical clustering of the transcripts that are significantly upregulated or downregulated by *E. coli* were generated (Figure 2A). Functional annotation analysis from Gene Ontology (GO) predictions revealed that the upregulated transcripts were significantly enriched in biological processes such as defense response to Gram-negative bacterium, positive regulation of Toll-like receptor signaling pathway, lipopolysaccharide-mediated signaling pathway, and positive regulation of NF-κB import into nucleus (Figure 2B). The downregulated genes were mainly associated with cell proliferation, cell death and metabolism process (Figure 2B). In Kyoto Encyclopedia of Genes and Genomes (KEGG) pathway analysis, the upregulated mRNAs were found to be mostly enriched in innate immune response pathways, including the tumor necrosis factor (TNF) signaling pathway, antigen processing and presentation, NF-κB signaling pathway, cytokine–cytokine receptor interaction, RIG-I-like receptor signaling pathway (RLR), Toll-like receptor (TLR) signaling pathway, and nucleotide-binding oligomerization domain (NOD)-like receptor signaling pathway (Figure 2C). The downregulated mRNAs were mostly enriched in the extracellular matrix–receptor (ECM-receptor) interaction pathway (15 DEGs) (Figure 2C).

GO enrichment and pathway analysis were not performed for DEGs induced by *S. aureus* particles due to the small number of genes. Functions of the DEGs are summarized in Table S2.

### 3.3. Searching for Nrf2 Target Genes in DEGs from E. coli-Stimulated Cells

**Figure 3:**
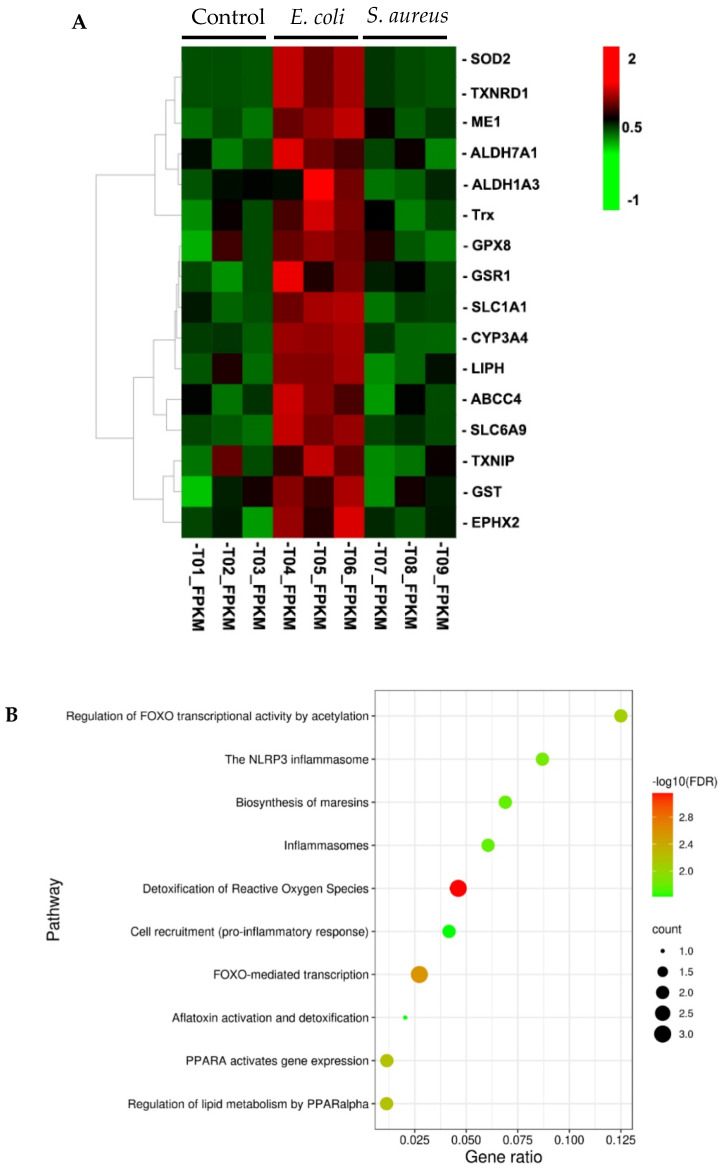
Reactome pathway analysis. (**A**) Hierarchical clustering of gene expression for the 16 selected Nrf2 target genes. Rows represent individual genes and columns represent the expression changes of replicate for each investigated group. Red color indicates relative over-expression, while green color indicates relative under-expression. (**B**) Bubble plots displaying over-represented Reactome pathways. y-axis, functional pathways; x-axis, the gene ratio of enriched relative to all genes in each pathway. The size of the bubble is proportional to the number of genes assigned to pathway and the color corresponds to the adjusted *p* value. A high FDR is represented by red, and a low value is represented by green.

Nrf2 pathway plays a vital role in maintaining cellular homeostasis and contributes to diverse cellular functions including inflammation [32]. We therefore asked whether the DEGs identified by RNA-seq analysis in the *E. coli*-treated samples versus normal contains previously reported Nrf2 regulated genes or not [14]. Since Nrf2 activation leads to upregulation of its downstream regulated genes, we focused on the upregulated DEGs for further analysis. Although the expression of the Nrf2 gene itself was not substantially changed, 16 genes downstream of Nrf2 were identified in the upregulated genes induced by *E. coli* (Table S3). Notably, all of these perturbations were absent in response to *S. aureus* stimulation (Figure 3A), suggesting that the ability of *E. coli* to potentiate an Nrf2 pathway response is not shared by *S. aureus*. To assess biological pathways overrepresented in these genes, we used online Reactome version (http://reactome.org/, 7 February 2020) with the “pathways for human” option for functional enrichment analysis [33]. Gene network analysis suggested that the upregulated Nrf2 target genes were enriched in gene sets with a focus on metabolism and the immune system (Figure S1). As shown in Figure 3B, detoxification of reactive oxygen species, the NLRP3 inflammasome, and cell recruitment (proinflammatory response) were significantly enriched in the upregulated Nrf2 target genes.

### 3.4. Validation of Selected Genes by qPCR

**Figure 4:**
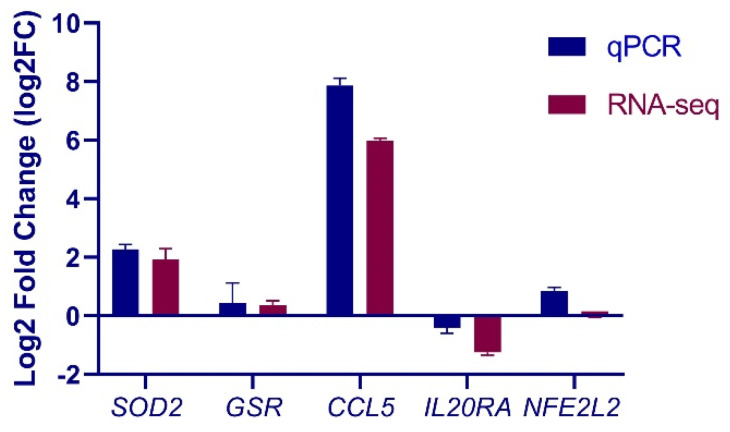
Real-time quantitative PCR (qPCR) validation of RNA sequencing (RNA-Seq) data. Relative mRNA abundance of *Nrf2*, Chemokine (C–C motif) ligand 5 (*CCL5*), glutathione reductase (*GSR1*), superoxide dismutase 2 (*SOD2*) and interleukin 20 receptor alpha (*IL20RA*) in *E. coli*-stimulated cells determined by RNA-seq and qPCR measurements. Results are expressed as log2FC over unstimulated control cells. Data are mean ± s.d. of triplicates.

To show the reliability of the DEG analysis of RNA-seq data, 4 genes showing differentially regulated expression in response to *E. coli* stimulation were quantified by using a SYBR-based real-time quantitative PCR (qPCR). More specifically, *SOD2* and *GRS1* were selected since, as mentioned above, they are transcriptionally regulated by Nrf2. Two additional genes, namely *CCL5* and *IL20RA*, represent the highly upregulated and moderately downregulated genes, respectively. The NFE2L2 (Nrf2) gene, although not differentially regulated, was also selected for validation. Our results showed that the qPCR expression patterns of these genes agreed with the RNA-seq results in terms of direction and magnitude, with the exception that the relative expression of *Nrf2* detected by qPCR, which was higher than by RNA-seq, but with a similar trend between the two methods (Figure 4). These results indicate a close correlation between qPCR and RNA-seq data.

### 3.5. Different Response of Nrf2 Pathway to E. coli and S. aureus

**Figure 5:**
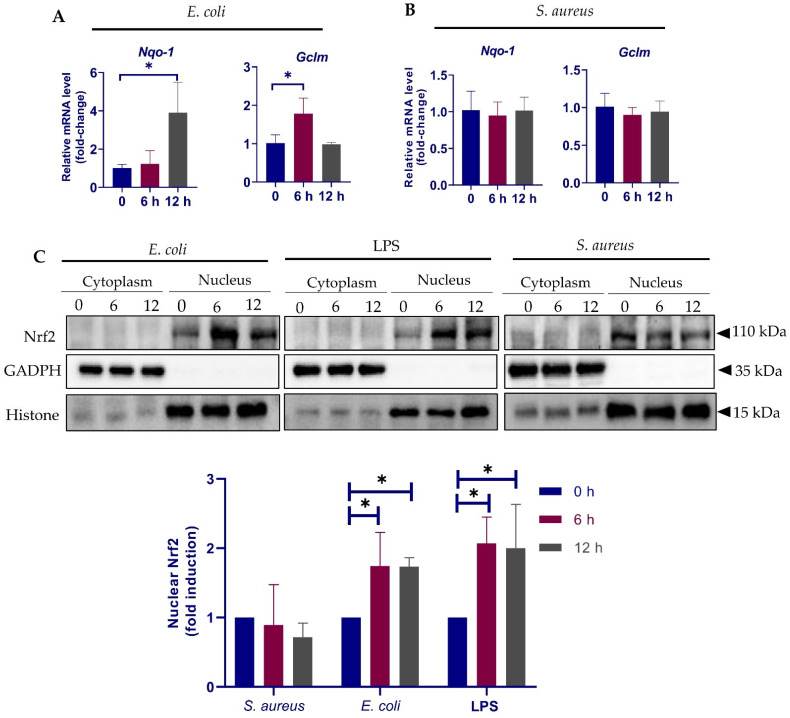

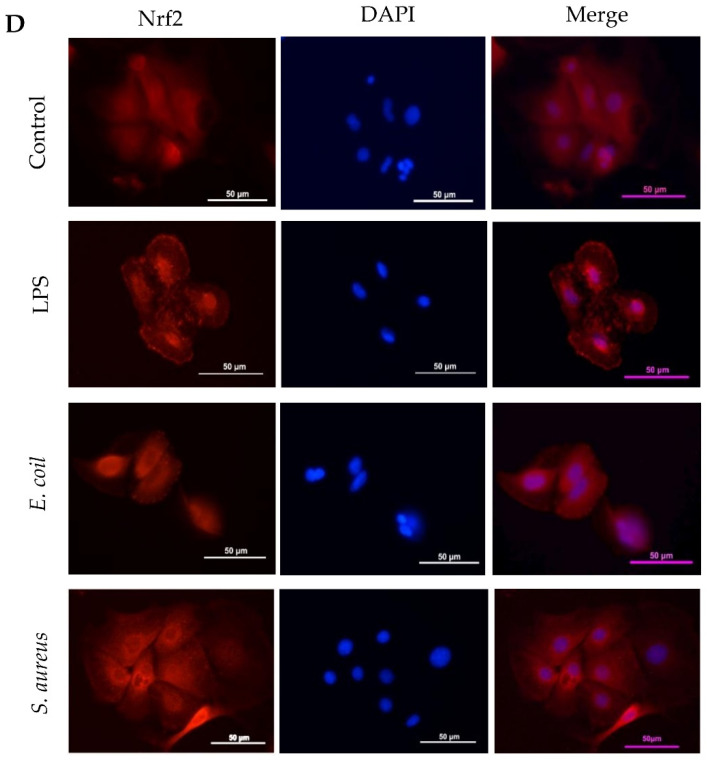
Response of Nrf2 pathway to *E. coli* and *S. aureus*. (**A**,**B**) pbMECs were treated with heat-killed *E. coli* (1 × 10^7^ particles/mL) (**A**) and *S. aureus* (1 × 10^7^ particles/mL) (**B**), respectively, for the indicated time. *Nqo-1* and *Gclm* mRNA expression was quantified by qPCR. Fold change is relative to control cells. Data are means ± s.d. of three triplicates and are representative of 3 separate experiments. (**C**) Cytoplasmic and nuclear proteins extracted from cells treated with *E. coli* (1 × 10^7^ particles/mL), *S. aureus* (1 × 10^7^ particles/mL) and lipopolysaccharides (LPS) (10 μg/mL) were analyzed by immunoblotting with anti-Nrf2. GADPH and Histone are shown as loading controls, respectively. Nuclear Nrf2 protein levels were determined with densitometry analyses after normalization to Histone. Bars are means ± s.d. of three triplicates and are representative of 3 separate experiments. * *p* < 0.05. (**D**) After treatment with *E. coli*, LPS or *S. aureus* for 12 h as described above, cells were subjected to immunofluorescent staining. Nrf2 was stained with an Alexa Fluor 594-conjugated secondary antibody. Cell nuclei were visualized by 4′,6-Diamidino-2-phenylindole dihydrochloride (DAPI). The images were obtained using inverted fluorescence microscopy.

To gain further insights into the earlier response of the Nrf2 pathway to *E. coli* and *S. aureus*, we treated the cells with *E. coli* and *S. aureus* particles for 6–12 h and detected by qPCR the expression of *Nqo-1* and *Gclm*, two notable targets of Nrf2 activation. In accordance with previous findings [34], *E. coli* stimulation resulted in an increased expression in both *Nqo-1* and *Gclm* (Figure 5A), suggesting Nrf2 activation. In contrast, no significant alteration in *Nqo-1* and *Gclm* was observed in *S. aureus*-treated cells by 12 h (Figure 5B). Considering that Nrf2 transactivates cytoprotective genes are only in the nucleus [32], we next determined by immunoblot analysis the Nrf2 protein levels in the cytoplasmic and nuclear extracts. Cells stimulated by *E. coli* O111:B4-derived crude lipopolysaccharide (LPS) was also analyzed to verify the results from *E. coli*. Although the predicted molecular weight of Nrf2 is ~55–65 kilodalton (kDa), it is believed that the biologically relevant band migrates between ~95 and 110 kDa on SDS-PAGE gel [35]. Indeed, two major bands between the 60 and 140 kDa molecular weight markers were present in our immunoblots (Figure S2) and only the ~110 kDa bands were selected for further analyses. In support of a previous report [36], Nrf2 was almost undetectable in the cytoplasmic fractions, regardless of the treatments (Figure 5C). Immunoblot analysis showed that *E. coli* particles induced an increase in Nrf2 accumulation in the nucleus, an effect that was reproducible in LPS-treated cells (Figure 5C). The data suggest that LPS-positive bacterium activates Nrf2 nuclear translocation, thereby initiating downstream transcription activities. In contrast, nuclear Nrf2 levels were slightly downregulated by *S. aureus* (Figure 5C). To confirm these results, we performed immunofluorescent staining of Nrf2, which demonstrated strongly increased Nrf2 signals in the nuclei of cells stimulated by LPS and *E. coli* particles as compared to normal cells; however, the nuclear immunofluorescence signals were weakened in response to *S. aureus* exposure (Figure 5D). Taken together, the results suggest that *E. coli* and *S. aureus* differentially regulate Nrf2 nuclear translocation in pbMECs.

### 3.6. Nrf2 Positively Regulates the Inflammatory Response in pbMECs

**Figure 6:**
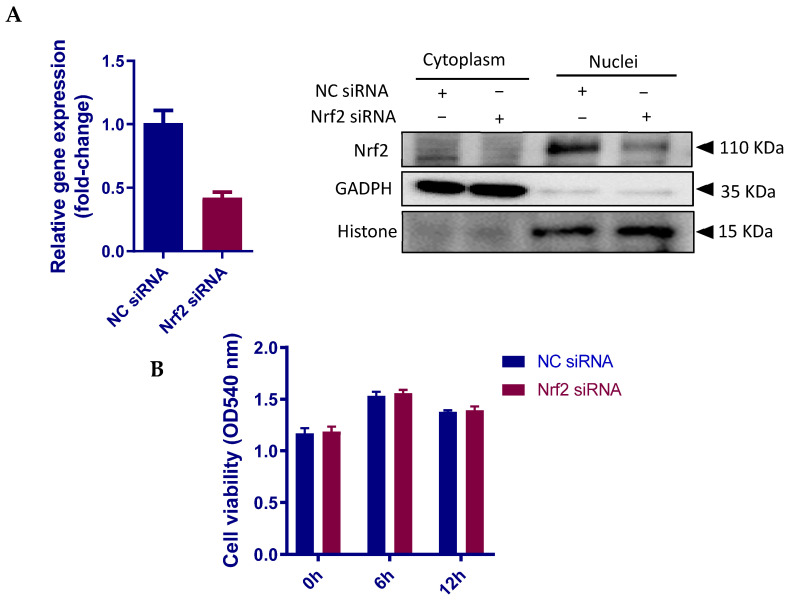

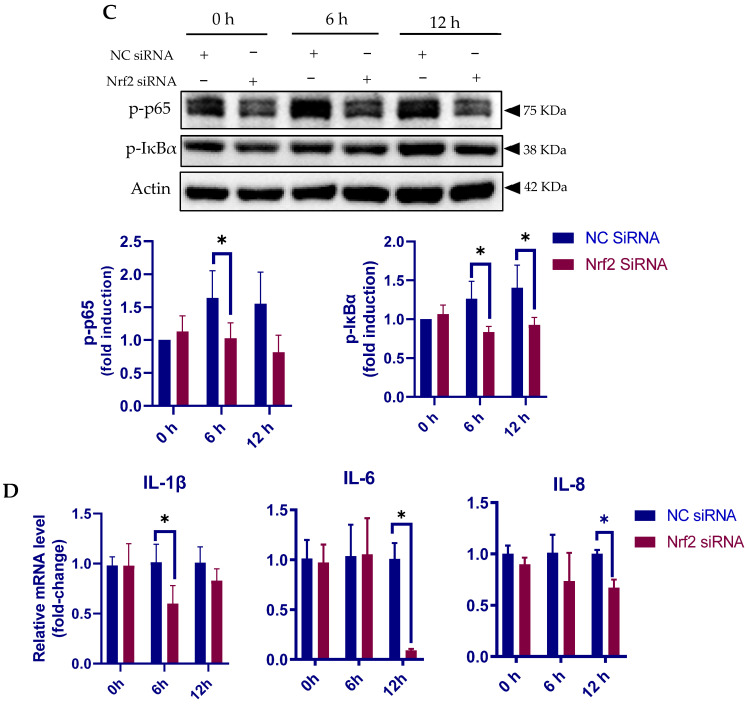
Nrf2 knockout attenuates LPS-induced inflammatory response in pbMECs. Cells were transfected with siRNA targeting Nrf2 or a negative control (NC) siRNA for 48 h, and the efficiency of Nrf2 knockdown was confirmed by RT-qPCR (left panel) and Western blotting (right panel). β-actin was used for the equal loading control (**A**). The cells were then incubated with LPS (10 μg/mL) for the indicated time. Cell viability was determined by MTT assay. The results are the mean ± s.d. of six replicates (**B**). The cells were then lysed with RIPA lysis buffer and subjected to immunoblot analysis against Nrf2, phosphor-IκBα (p-IκBα) and NF-κB p65 (p-p65) by using anti-Nrf2, anti-pIκBα, and anti-pNF-κB p65 antibodies, respectively. β-actin was used for the equal loading control. p-p65 and p- IκBα levels were quantified with densitometry analyses after normalization to Actin. Bars are means ± s.d. of three triplicates and are representative of 3 separate experiments (**C**). Total RNAs were prepared and subjected to qPCR analyses for the mRNA levels of *IL-1β*, *TNF-α*, *IL-6* and *IL-8*. The results are the mean ± s.d. of three replicates and are representative of 3 separate experiments (**D**). * *p* < 0.05.

Multiple studies have suggested that Nrf2 confers protection against inflammation [37]. However, Nrf2 knockout in murine fibroblasts has been shown to suppress p50 and p65 subunits of NF-κB [38], a master regulator of innate immune responses. We next used an LPS-stimulated inflammatory cell model to verify the contribution of Nrf2 in the inflammatory response of pbMECs. Nrf2 siRNA was employed to downregulate the gene and protein expressions (Figure 6A). No significant effect of Nrf2 knockdown on cell viability was determined before or after LPS stimulation (Figure 6B). As shown in Figure 6C, siRNA-mediated Nrf2 knockdown attenuated LPS-induced phosphorylation of both IκBα and NF-κB p65 as compared to negative control siRNA (NC-siRNA). We also measured mRNA levels by qPCR of genes encoding proinflammatory cytokines interleukin (IL)-1β, IL-6 and IL-8. Corresponding to the immunoblotting data, LPS-induced transcriptional upregulation of the cytokines was suppressed by Nrf2 siRNA (Figure 6D). The data suggest that Nrf2 positively regulates the innate immune response of bovine mammary epithelial cells.

**Figure 7:**
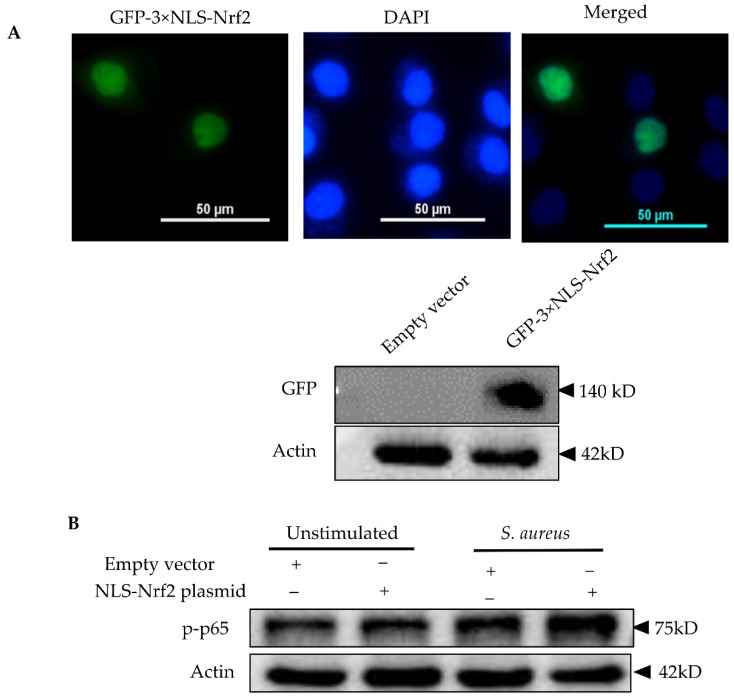

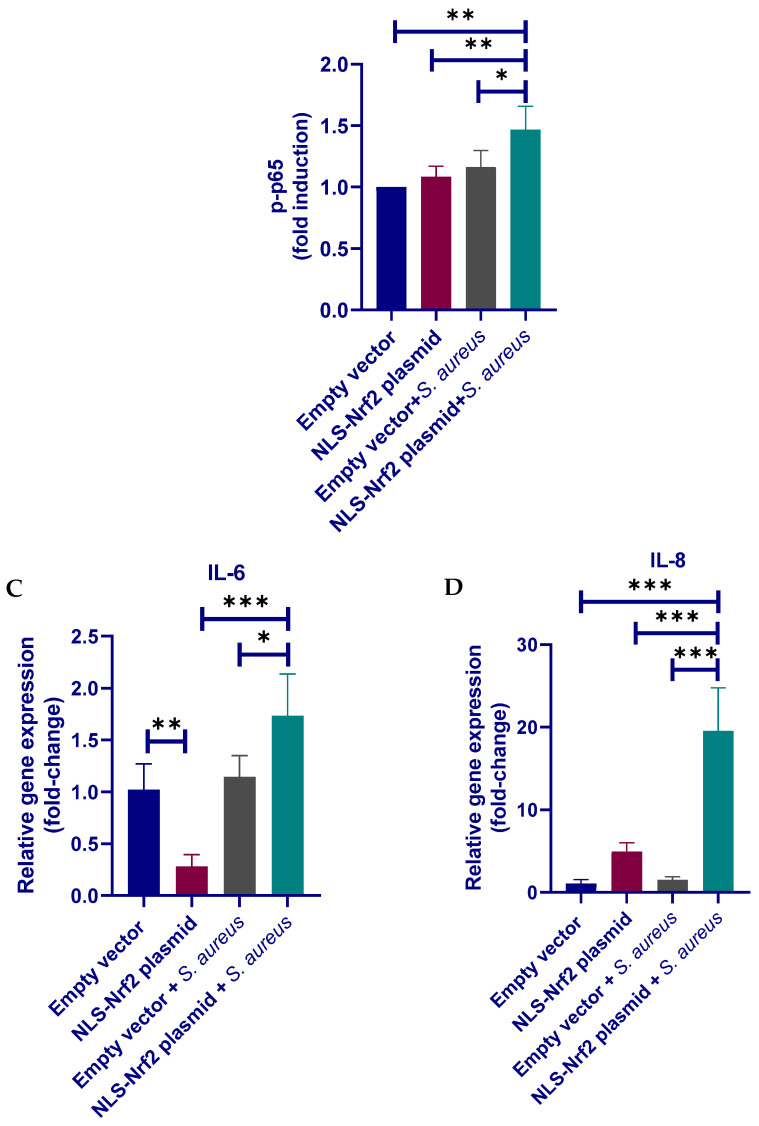
Evaluation of Nrf2 overexpression on the inflammatory responses of pbMECs to *S. aureus*. (**A**) Confirmation of Nrf2 overexpression. Cells were transfected with a GFP-expressing Nrf2 plasmid containing tripartite nuclear localization signal (NLS-Nrf2) for 24 h. GFP fluorescence was determined under a microscope and whole-cell extracts were analyzed by immunoblotting with anti-GFP. (**B**) Evaluation of the effect of Nrf2 overexpression on NF-κB p65 activation in response to *S. aureus*. The cells transfected with GFP-expressing Nrf2 plasmid were incubated with *S. aureus* for 6 h. Whole-cell extracts were analyzed by immunoblotting with anti-phospho NF-κB p65 antibody (p-p65). β-actin was used for the equal loading control. p-p65 levels were determined with densitometry analyses after normalization to Actin. Bars are means ± s.d. of three triplicates and are representative of 3 separate experiments. (**C**,**D**) Evaluation of the effect of Nrf2 overexpression on the expression of pro-inflammatory cytokines was induced by *S. aureus*. The cells transfected with GFP-expressing Nrf2 plasmid were incubated with *S. aureus* for 6 h. Total RNAs were prepared and subjected to qPCR analyses for the mRNA levels of *IL-6* and *IL-8*. The results are the mean ± s.d. of three replicates and are representative of 3 separate experiments (**D**). * *p* < 0.05, ** *p* < 0.01 and *** *p* < 0.001.

Based on these findings, we postulated that Nrf2 overexpression might enhance the cytokine responses of mammary epithelial cells to *S. aureus*. In order to overexpress Nrf2, we first transfected pbMECs with a pEGFP-C3-Nrf2 plasmid, but we failed to determine neither GFP fluorescence nor GFP protein in the transfected cells, although the relative Nrf2 mRNA abundance was increased more than 1000-fold of the control cells (data not shown). It is thus our belief that exogenous Nrf2 protein was rapidly degraded in the cytoplasm after translation. In order to reduce exogenous Nrf2 degradation, we next transfected the cells with a GFP-expressing Nrf2 plasmid containing tripartite nuclear localization signal (NLS-Nrf2) to ensure the nuclear import. Fluorescence of GFP was detectable in nuclei 24 h after transfection and increased accumulation of GFP-Nrf2 was confirmed by Western blot analysis (Figure 7A). However, only limited number of cells (~20%) displayed GFP fluorescence. Nevertheless, treatment of Nrf2 plasmid increased NF-κB activity following *S. aureus* stimulation as compared to the mock plasmid (Figure 7B). Interestingly and unexplainably, although Nrf2 plasmid downregulated *IL-6* expression under normal conditions, which is in agreement with a previous report using macrophage [37], a moderate increase in IL-6 mRNA was determined in cells bearing Nrf2 plasmid as compared to mock plasmid following *S. aureus* stimulation (Figure 7C). In contrast to *IL-6*, and in line with a previous report [39], Nrf2 plasmid induced *IL-8* gene expression in unstimulated cells, and this effect was further augmented in the presence of *S. aureus* (Figure 7D). Similarly, the expression of *IL-6* and *IL-8* in response to *S. aureus* was augmented by tBHQ (Figure S5). Taken together, contrary to the widely accepted view that Nrf2 has an anti-inflammatory effect [37], our findings suggest that Nrf2 induction is required for pbMECs to mount an efficient inflammatory response.

### 3.7. S. aureus-Induced Nrf2 Activation Impairment Is Not Due to Inadequate ROS Production

**Figure 8:**
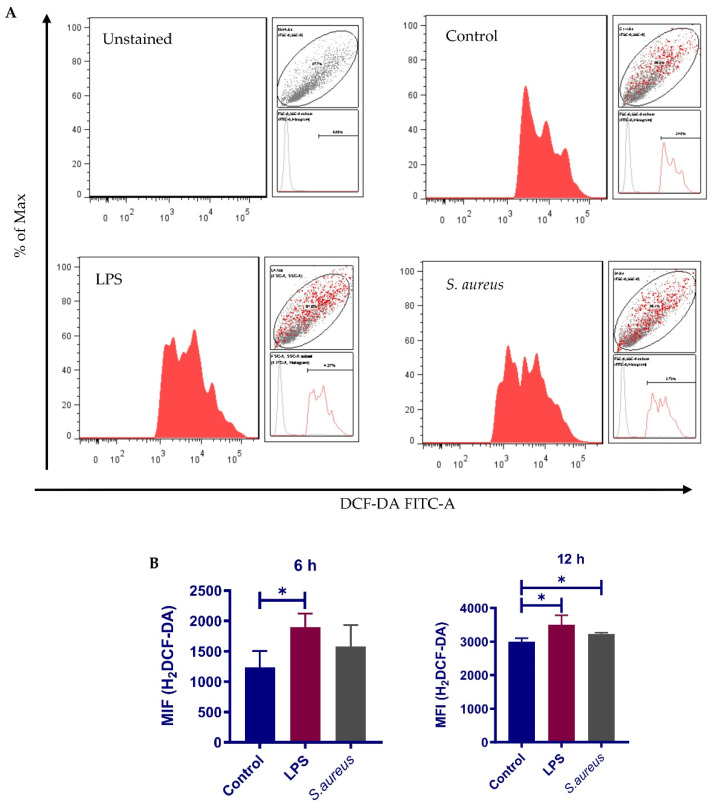
Determination of the production of ROS using the 2’,7’-dichlorofluorescin diacetate (DCF-DA) probe. pbMECs were treated with LPS (10 μg/mL) or *S. aureus* (1 × 10^7^ particles/mL) for 6 and 12 h, followed by incubation with serum-free medium containing H2DCF-DA (20 µM) for 30 min. 2,7-dichlorofluorescein (DCF) fluorescence was measured using flow cytometry. (**A**) Representative flow cytometry histograms showing DCF-DA staining of cells treated with LPS or S. aureus for 12 h. (**B**) Mean fluorescence intensities (MIF) are shown as bar graph. Data are mean ± s.d. (*n* = 3). * *p* < 0.05.

Under normal conditions, Nrf2 forms a complex with Keap1 (kelch-like ECH-associated protein 1), which functions as a key repressor of Nrf2, in cytoplasm in the absence of an activator. Keap1-Nrf2 pathway is activated when cells are exposed to oxidative and electrophilic stresses of both exogenous and endogenous origins [12]. To demonstrate that impairment of Nrf2 activation in response to *S. aureus* is not due to inadequate ROS production, intracellular ROS level was measured by using a 2’,7’-dichlorofluorescin diacetate (DCFH-DA) probe. In line with an earlier study, pbMEC exposure to *S. aureus* particles triggered a time-dependent increase in ROS, which was comparable to that in LPS-treated cells (positive control) by 12 h (Figure 8). To rule out the possibility that the Nrf2 pathway in pbMECs is not sensitive to oxidative stress, we used the LPS-stimulated cell model to determine the effect of N-acetylcysteine (NAC), an ROS scavenger, on Nrf2 activation. qPCR analysis showed that treatment with 10 mM NAC led to a significant downregulation in the expression of Nrf2 target genes, such as *Nqo-1*, *Gclm*, *Sod1* and *p62*, as compared to mock-treatment (Figure S2). Considering that cells treated with *S. aureus* also demonstrated increased ROS production but had impaired Nrf2 activation, we wondered if *S. aureus* could induce Nrf2 degradation. To this end, we pretreated the cells with tert-butylhydroquinone (tBHQ), a powerful Nrf2 inducer [40,41], for 2 h to induce robust Nrf2 activation prior to *S. aureus* exposure (Figure S3). If *S. aureus* induces Nrf2 degradation, one can expect a decline in the transactivation activity of Nrf2. Indeed, we found that the induction of Nrf2 target genes by tBHQ was not affected by *S. aureus* particles (Figure S4), suggesting that *S. aureus*-induced impairment of Nrf2 activation is not due to increased Nrf2 degradation.

### 3.8. Both S. aureus and E. coli Upregulates p62/SQSTM1 Levels but Differentially Modify p62/SQSTM1 Phosphorylation

**Figure 9:**
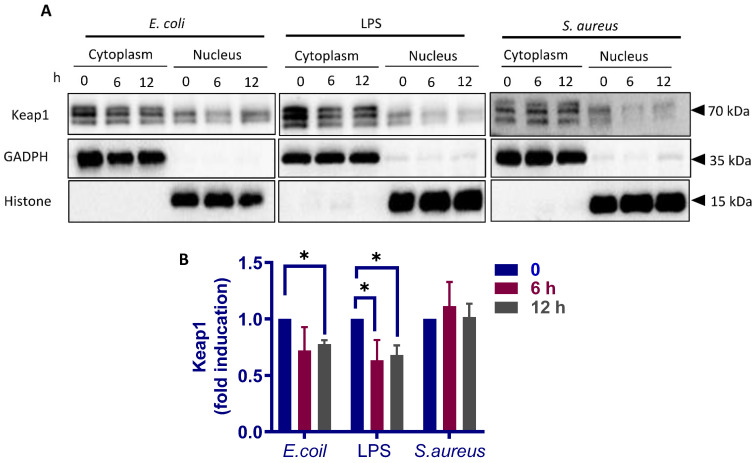
*E. coli* and *S. aureus* differentially regulate Keap1 degradation. (**A**) Cytoplasmic and nuclear proteins were extracted from cells treated with *E. coli* (1 × 10^7^ particles/mL), *S. aureus* (1 × 10^7^ particles/mL) and lipopolysaccharides (LPS) (10 μg/mL) and were analyzed by immunoblotting with anti-Keap1. GADPH and Histone are shown as loading controls, respectively. (**B**) A chart bar shows Keap1 levels determined with densitometry analyses after normalization to GADPH. Data are representative of three triplicates and are representative of 3 separate experiments. * *p* < 0.05.

We next questioned whether *E. coli* and *S. aureus* differentially modulates Keap1 degradation in pbMECs. Immunoblot analysis showed that both *E. coli* and LPS triggered a significant reduction in cytoplasmic Keap1. By contrast, the levels of cytoplasmic Keap1 was not changed in response to *S. aureus* (Figure 9). A small amount of Keap1 was also determined in the nuclear fractions (Figure 9A), possibly due to the cytoplasmic contamination. Therefore, this portion was not taken into consideration. Taken together, the results suggest that Keap1 degradation is involved in *E. coli*-induced Nrf2 activation in pbMECs, and that a lack of such a mechanism may account for the impairment of Nrf2 activation during *S. aureus* infection.

**Figure 10:**
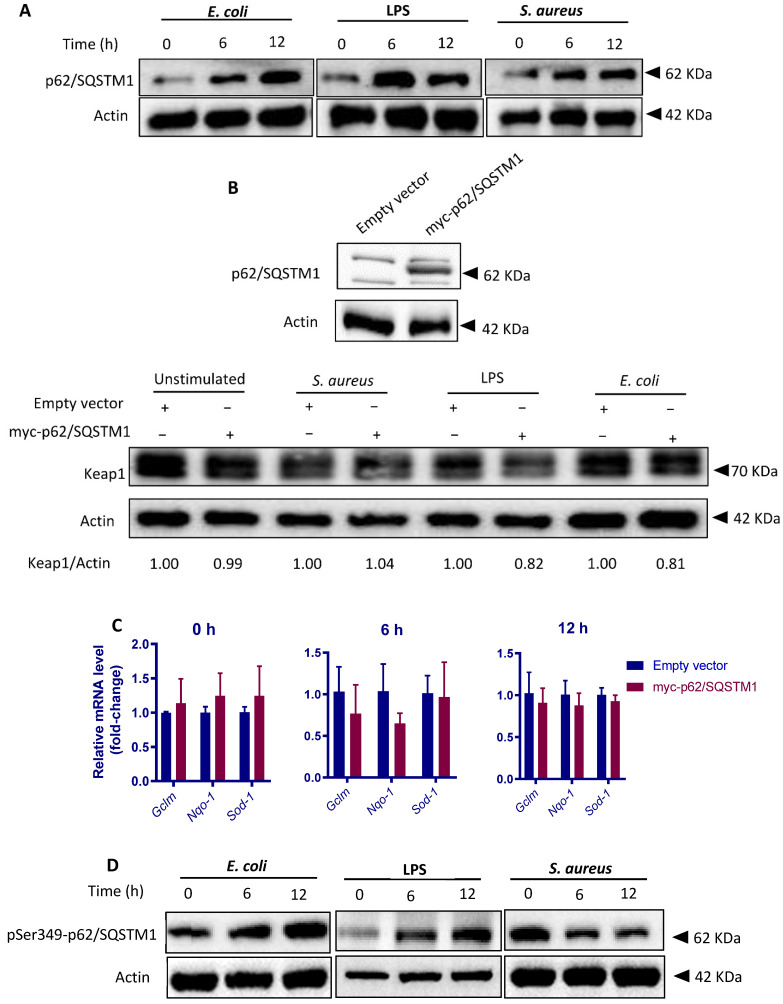
*S. aureus* and *E. coli* differentially modify p62/ SQSTM1 phosphorylation**.** (**A**) Evaluation of the p62/SQSTM1 protein levels in pbMECs. Cells were treated with heat-killed *E. coli* (1 × 10^7^ particles /mL), LPS (10 μg/mL) or *S. aureus* (1 × 10^7^ particles/mL) for the indicate time. Whole-cell extracts were analysed by immunoblotting with anti-p62/SQSTM1. β-actin is shown as a loading control. The data are representative of 2 independent experiments. (**B**) Evaluation of the p62/SQSTM1-dependent Keap1 degradation. pbMECs were transfected with either mock (empty vector) or myc-p62/SQSTM1 plasmid. Forty-eight hours post-transfection, the cells were treated heat-killed *E. coli* (1 × 10^7^ particles /mL), LPS (10 μg/mL) or *S. aureus* (1 × 10^7^ particles/mL) for a further 6 h. Whole-cell extracts were analysed by immunoblotting with anti-Keap1. β-actin is shown as a loading control. The data are representative of 2 independent experiments. (**C**) Evaluation of Nrf2 activation in cells with p62/SQSTM1 overexpression in response to *S. aureus*. Mock-transfected and p62/SQSTM1 plasmid-transfected cells were treated as indicated above. Total mRNA was extracted and RT-qPCR was performed to measure the mRNA levels of Nrf2 target genes *Ho-1*, *Glcm*, *Nqo-1*, and *Sod1*. Data presented are the mean ± s.d. of three triplicates and are representative of 3 separate experiments. (**D**) Evaluation of the effect of *E. coli*, *S. aureus* and LPS on phosphorylation of p62/SQSTM1 at Ser349 (pSer349-p62/SQSTM1). pbMECs were treated with heat-killed *E. coli* (1 × 10^7^ particles /mL), LPS (10 μg/mL) or *S. aureus* (1 × 10^7^ particles/mL) for the indicated time. Whole-cell extracts were analysed by immunoblotting with anti-pSer349-p62/SQSTM1. β-actin is shown as a loading control. The data are representative of 2 independent experiments.

To further clarify the mechanisms by which *E. coli* and *S. aureus* differentially regulate Keap1 degradation, we focused on p62/sequestosome 1 (SQSTM1), a stress sensor that has been shown to disrupt the Nrf2-Keap1 complex by competing with Keap1 for Nrf2 binding and to target Keap1 for autophagic degradation [27]. This mechanism of activating Nrf2 is non-canonical, in contrast to the previously described canonical pathway by which Nrf2 is activated via oxidative modifications in the cysteine residues in Keap1 [42,43]. LPS-induced Nrf2 activation has been previously attributed to p62/SQSTM1-mediated Keap1 degradation [44]. Consistent with this study, p62/SQSTM1 levels were significantly upregulated by *E. coli* as well as LPS. Similarly, the levels of p62/SQSTM1 protein were also upregulated following *S. aureus* treatment (Figure 10A). The results led us to question whether increased amount of p62/SQSTM1 plays a role in Keap1 turnover in our cell models. Therefore, we treated the cells with an expressing plasmid encoding p62/SQSTM1. Overexpression of p62/SQSTM1 was confirmed by Western blot analysis (Figure 10B). As expected, p62/SQSTM1 overexpression enhanced Keap1 turnover in the presence of *E. coli*; however, this effect was not evident when cells were exposed to *S. aureus* (Figure 10B). In support of this result, the transcription of Nrf2 target genes in p62/SQSTM1-overexpressing cells was not activated by *S. aureus* (Figure 10C). The data suggest that *S. aureus* dampens p62/SQSTM1-mediated Keap1 turnover. To this point, we were interested to see the level of phosphorylation of p62/SQSTM1 at Ser349 (Ser351 in mouse), which rises affinity of p62/SQSTM1 for Keap1 and contributes subsequent Nrf2 activation [27,45]. Western blot analysis showed that Ser349-phosphorylated p62/SQSTM1 (pS349-p62) levels were markedly increased in response to *E. coli* stimulation; by contrast, *S. aureus* particles induced a significant reduction in pS349-p62 level in a time-dependent manner (Figure 10D). Taken together, the results strongly suggest that *S. aureus* perturbs p62/SQSTM1-mediated Keap1 degradation through perturbing phosphorylation of p62/SQSTM1.

## 4. Discussion

A number of studies have shown the different immune responses of the bovine mammary gland to distinct mastitis-causing pathogens [9,25,46]. Although the underlying molecular mechanisms are not fully understood, recent studies have repeatedly demonstrated that Gram-positive bacteria fail to activate pathogen receptor-derived activation of IκB/NF-κB signaling as compared to Gram-negative bacteria [47,48]. Increasing evidence suggests that MECs in culture are able to model the key aspects of the immune response profile of pathogen species-specific mastitis [20,49,50]. Since it will take a longer time (24 h) for *S. aureus* than for *E. coli* to achieve the maximum impact on gene expression in host cells, and genes encoding cytokines and transcription regulators (e.g., NF-κB) are constantly regulated by *E. coli* for 24 h [30], we challenged our pbMECs with *E. coli* and *S. aureus*, respectively, for 24 h before transcriptome analysis. Our data showed that challenging MECs with *E. coli* particles resulted in >1400 DEGs, while the *S. aureus* challenge altered the expression of only 16 genes. However, in another study, *S. aureus* challenge has been to shown to regulate more than 100 genes in pbMECs [30]. The discrepancy could be due to the difference in *S. aureus* strains investigated, since significant differences in host response to bovine-associated lineages have been previously reported [51]. *E. coli* induced the expression of a wealth of immune genes involved in inflammatory response signaling pathways such as the TLR signaling pathway, NOD-like receptor signaling pathway, and NF-κB signaling pathway. On the contrary, none of these pathways were significantly affected by *S. aureus* particles. This is in agreement with other transcriptional profiling studies demonstrating that *E. coli* stimulation strongly upregulates the genes involved in the NF-κB pathway, whereas *S. aureus* does not [21,30,47]. Overall, our results support the notion that a lack of NF-κB activation accounts for the impaired immune response elicited by *S. aureus* [50].

Although extensively studied, the mechanisms through which *S. aureus* inhibits NF-κB activation in bovine mammary epithelial cells remains largely unclear. The Nrf2 signaling pathway plays a pivotal role in defense against oxidative stress and toxic insults and acts as a critical regulator of the innate immune response [37,52]. The distinct inflammatory response of pbMECs to *E. coli* and *S. aureus* led us to suggest that the Nrf2 pathway is differently regulated by the two pathogens. In support of this hypothesis, transcriptome differences revealed a distinct expression pattern of Nrf2 target genes in pbMECs challenged with *E. coli* and *S. aureus*, in that multiple Nrf2 target genes were induced by *E. coli*, while none of the genes dysregulated by *S. aureus* was linked to the Nrf2 pathway. In addition, Gene network analysis and KEGG pathway analysis results demonstrated that the upregulated Nrf2 target genes in response to *E. coli* particles are mainly involved in anti-oxidant and immune response. In accordance with the transcriptome analysis, Western blot analysis validated that *E. coli* was induced whereas *S. aureus* inhibited Nrf2 nuclear accumulation. No previous studies have evaluated the effect of *S. aureus* on Nrf2 induction in pbMECs. However, in a recent study, LPS has been shown to inhibit Nrf2 activation in Mac-T [53], a bovine mammary cell line obtained by stable transfection of mammary alveolar cells with simian virus-40 (SV-40) and large T-antigens [54]. Notably, there is a marked difference in the transcriptome signatures between mammary tissue and Mac-T cells [55], which might explain the difference in the Nrf2 response between pbMECs and Mac-T.

Several studies have demonstrated that Nrf2 produces an anti-inflammatory effect [37,56,57]. In stark contrast, the present study demonstrated that Nrf2 is indispensable for pbMECs to mount an efficient immune response. Supporting this notion is that Nrf2 knockdown significantly diminished LPS-induced NF-κB activation and transcription of pro-inflammatory cytokines. In support of our findings, Nrf2 is proved to positively regulate hepatic IL-6 expression [58] and NF-κB production [38]. Moreover, Nrf2 was found to be essential for cholesterol crystal-induced inflammasome activation and IL-1 production in vascular cells [59]. In a recent study, Nrf2 and NF-κB are found to be regulated in the same direction in bovine granulosa cells during lead toxicity [60]. Thus, it appears that Nrf2 activation may enhance or attenuate inflammatory response, depending on the cell type/tissue context and stimuli. Considering the pro-inflammatory effect of Nrf2 in pbMECs, we could conclude that the weak inflammatory response of pbMECs to *S. aureus* is associated with impaired Nrf2 activation. Indeed, we did observe an enhanced transcription of pro-inflammatory cytokines in response to *S. aureus* in cells with Nrf2 overexpression.

Oxidative stress is a critical factor driving Nrf2 to detach from Keap1 and to subsequently translocate into the nucleus. Under basal conditions, Keap1 binds to the Neh2 domain of Nrf2 in the cytoplasm and targets Nrf2 for ubiquitination and proteasomal degradation. Upon oxidative stress, Nrf2 dissociates from Keap1 and translocates into the nucleus, where it transactivates several cytoprotective genes [26,61,62]. In the present study, we observed an ROS-dependent Nrf2 activation in pbMECs in response to LPS; in stark contrast, upon *S. aureus* challenge, pbMECs displayed increased intracellular ROS production but had reduced Nrf2 nuclear accumulation. Given that Nrf2 plays a major role in cellular defense against oxidative stress, and that *S. aureus* has the ability to encounter ROS [63], our results might explain why *S. aureus* infection usually causes severe damage in bovine udder tissue. In addition, a recent study has demonstrated that ROS produced by host cells coerce *S. aureus* into an antibiotic-tolerant state [64].

We next sought to clarify how *S. aureus* impairs Nrf2 activation. Although *S. aureus* produces a variety of proteins that can activate host zymogens targeting host components [65], we did not observe a direct impact of *S. aureus* on Nrf2 degradation by using a tBHQ-stimulated cell model. Indeed, we found that Keap1 protein was degraded in pbMECs in response to *E. coli* or LPS, but not following incubation with *S. aureus*, suggesting a high likelihood that *S. aureus* impairs Keap1 inactivation machinery. We next focused on p62/SQSTM1, a selective autophagy receptor that has been shown to play a central role in Nrf2 activation under oxidative stress through regulating autophagic degradation of Keap1 [27,42,66]. In agreement with a previous study [67,68], we observed an increase in p62/SQSTM1 protein in response to *S. aureus*, resembling upregulation of p62/SQSTM1 protein following LPS or *E. coli* exposure. Taken together, the data suggest that *S. aureus* impairs p62/SQSTM1-mediated Keap1-degradation through autophagy. Indeed, in contrast to *E. coli*, we observed that *S. aureus* failed to induce Keap1 turnover even in cells bearing p62/SQSTM1-expressing plasmid. We next determined the level of Ser349-phosphorylated p62/SQSTM1, which has been previously shown to play a major role in p62/SQSTM1-Keap1 interaction through modulating the binding affinity of p62/SQSTM1 for Keap1 [27,45]. As expected, *S. aureus* challenge resulted in a marked reduction in Ser349-phosphorylated p62/SQSTM1. In contrast, LPS or *E. coli* stimulation had the opposite effect on phosphorylated p62/SQSTM1. Taken together, our results suggest that p62/SQSTM1-binding dependent Keap1 inactivation is impaired by *S. aureus*. The underlying mechanisms by which *S. aureus* manipulates p62/SQSTM1 phosphorylation remain unclear. Recent studies have suggested that TLR signaling is involved in p62/SQSTM1-mediated Keap1 reduction [44,69]. Given that *E. coli* strongly stimulates TLR pathway activation in MECs, whereas *S. aureus* induces only a slight transient activation of TLR2 [50], a linkage between dysregulated TLR signaling and impaired phosphorylation of Ser349 p62/SQSTM1 in *S. aureus*-stimulated cells could be suggested.

## 5. Conclusions

In summary, this work demonstrates a pathogen species-specific response of the Nrf2 pathway in bovine mammary epithelial cells. We show for the first time that *S. aureus* impairs Nrf2 activation as compared to *E. coli* and that Nrf2 overexpression could augment *S. aureus*-stimulated inflammatory response. We have also identified molecular mechanisms by which *S. aureus* impairs Nrf2 activation. It is suggested that impairment of Nrf2 activation contributes to the weak inflammatory response in *S. aureus* mastitis.

## Data Availability

All data analyzed during this study are included in this published article and its Supplementary Information Flies.

## Supplementary Materials

The following are available online at https://www.mdpi.com/article/10.3390/cells10123426/s1, Figure S1: Enrichment map of Reactome pathways enriched in upregulated Nrf2 target genes; Figure S2: Determination of biologically relevant band of Nrf2 on SDS-PAGE gel; Nrf2 activation by LPS is ROS-dependent, Figure S3: Nrf2 activation by LPS is ROS-dependent; Figure S4: The effect of *S. aureus* on tBHQ-induced Nrf2 activation. Figure S5: The effect of tBHQ on the proinflammatory cytokine transcription in response to *S. aureus*. Table S1: Sequences of oligonucleotide primers used for real-time quantitative PCR analysis; Table S2: Dysregulated genes in response to *S. aureus* stimulation compared to unstimulated control cells; Table S3: Dysregulated Nrf2 downstream target genes in response to *E. coli* and *S. aureus* stimulation compared to unstimulated control cells; Additional file 1: Upregulated genes in response to *E. coli* demonstrated by RNA-seq; Additional file 2: Downregulated genes in response to *E. coli* demonstrated by RNA-seq.
