# Supplementary material for: Escherichia coli and Staphylococcus aureus Differentially Regulate Nrf2 Pathway in Bovine Mammary Epithelial Cells: Relation to Distinct Innate Immune Response
Source: Cells. 2021 Dec 6;10(12):3426. doi: 10.3390/cells10123426 (PMC8700232; doi:10.3390/cells10123426)
Supplement: Supplementary file 1 [file cells-10-03426-s001.zip › cells-1449855-supplementary/supplementary files/Fig. S1.pdf]

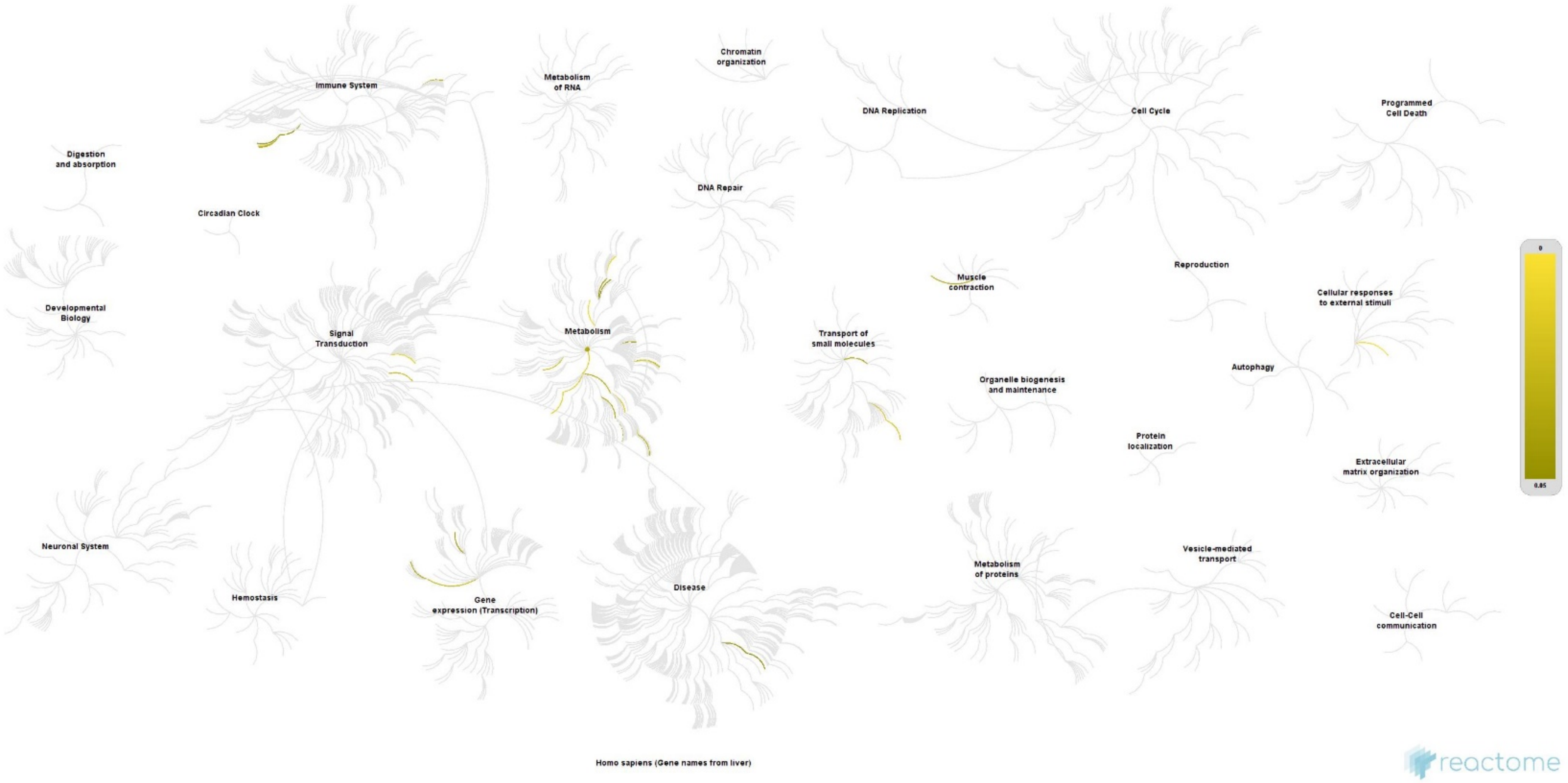

Fig. S1. Enrichment map of Reactome pathways enriched in upregulated Nrf2 target genes. Pathways overrepresented are represented in yellow and pathways not significantly overrepresented are represented in gray. Significant enrichment of a pathway was defined as FDR <0.05.
