# Supplementary material for: Escherichia coli and Staphylococcus aureus Differentially Regulate Nrf2 Pathway in Bovine Mammary Epithelial Cells: Relation to Distinct Innate Immune Response
Source: Cells. 2021 Dec 6;10(12):3426. doi: 10.3390/cells10123426 (PMC8700232; doi:10.3390/cells10123426)
Supplement: Supplementary file 1 [file cells-10-03426-s001.zip › cells-1449855-supplementary/supplementary files/Fig. S2.pdf]

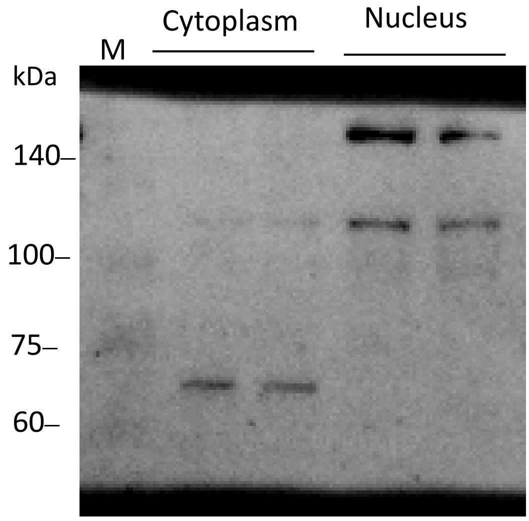

Fig. S2. Determination of biologically relevant band of Nrf2 on SDS-PAGE gel. Cytoplasmic and nuclear proteins were extracted from normal primary bovine mammary epithelial cells (bMEC) and subjected to Western blot analysis with an anti-Nrf2 antibody. One band with molecular weight of ~65 kDa in the cytoplasmic fractions and one of ~110 kDa in the nuclear fractions were determined. Two different samples were used.
