# Supplementary material for: Escherichia coli and Staphylococcus aureus Differentially Regulate Nrf2 Pathway in Bovine Mammary Epithelial Cells: Relation to Distinct Innate Immune Response
Source: Cells. 2021 Dec 6;10(12):3426. doi: 10.3390/cells10123426 (PMC8700232; doi:10.3390/cells10123426)
Supplement: Supplementary file 1 [file cells-10-03426-s001.zip › cells-1449855-supplementary/supplementary files/Fig. S3.pdf]

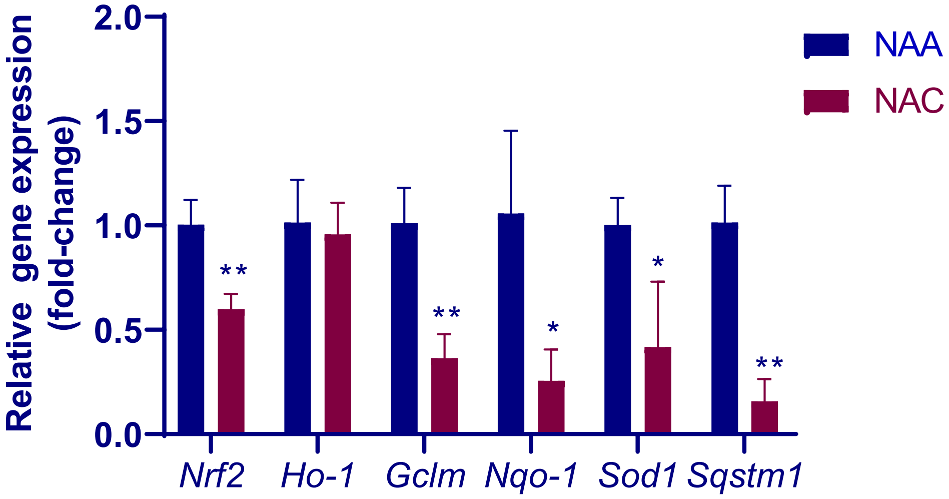

Fig. S3. Nrf2 activation by LPS is ROS-dependent. Cells were pretreated with NAA or NAC at 20 mM for 1 h and then incubated with LPS (10 µg/mL) for further 6 h. Total RNAs were prepared and subjected to qPCR for determining the mRNA levels of *Nrf2*, *Ho-1*, *Gclm, Nqo-1, Sod1* and *Sqstm1*. The results are the mean ± s.d. of three replicates and are representative of 3 separate experiments. **P* < 0.05 and ** *P* < 0.01 compared to NAA.
