# Supplementary material for: Escherichia coli and Staphylococcus aureus Differentially Regulate Nrf2 Pathway in Bovine Mammary Epithelial Cells: Relation to Distinct Innate Immune Response
Source: Cells. 2021 Dec 6;10(12):3426. doi: 10.3390/cells10123426 (PMC8700232; doi:10.3390/cells10123426)
Supplement: Supplementary file 1 [file cells-10-03426-s001.zip › cells-1449855-supplementary/supplementary files/Fig. S4.pdf]

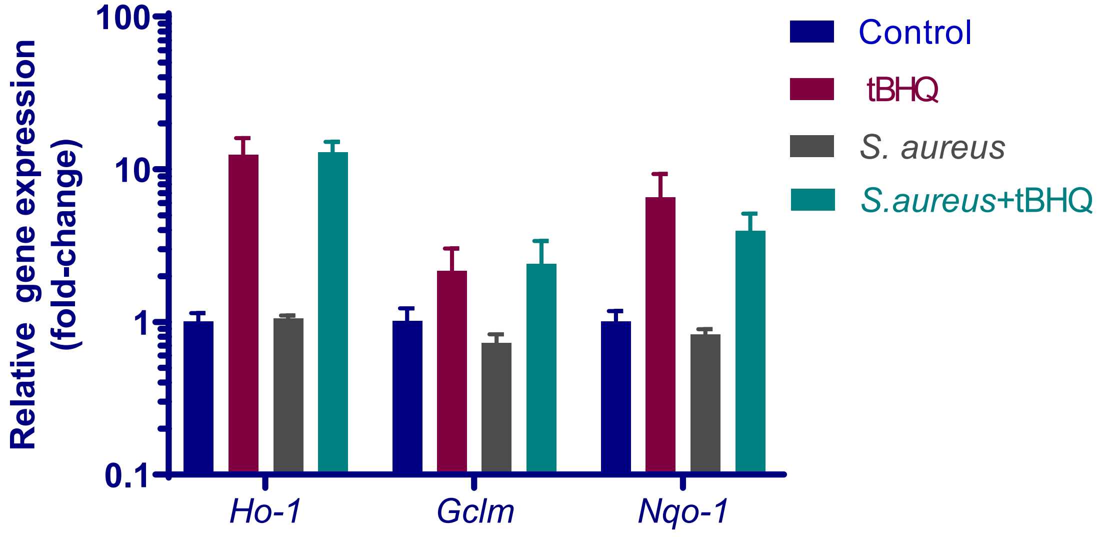

Fig. S4. The effect of *S.aureus* on tBHQ-induced Nrf2 activation. Cells were pretreated with *tert*-butylhydroquinone (tBHQ) at 50 μM for 1 h followed by incubation with *S. aureus* (1×10^7 particles/mL) for further 6 h. Total RNAs were prepared and subjected to qPCR analyses for determining mRNA levels of *Ho-1*, *Gclm* and *Nqo-1*. The results are the mean ± s.d. of three replicates.
