# Supplementary material for: Escherichia coli and Staphylococcus aureus Differentially Regulate Nrf2 Pathway in Bovine Mammary Epithelial Cells: Relation to Distinct Innate Immune Response
Source: Cells. 2021 Dec 6;10(12):3426. doi: 10.3390/cells10123426 (PMC8700232; doi:10.3390/cells10123426)
Supplement: Supplementary file 1 [file cells-10-03426-s001.zip › cells-1449855-supplementary/supplementary files/Table S1.pdf]

Table S1 Sequences of oligonucleotide primers used for real-time quantitative PCR analysis

Gene	Gen Bank Accession	Primer sequences（5'→3'）	Amplicon size (bp)	Annealing temperature (°C)	Validation	
					Efficiency	R^2
PGK1	NM_001034299	F: TGCAGCTGAATTGCCAAGATG<br>R: CAGCCTTGATCCTCTGGTTGT	145	60	92%	0.999
RPL19	XM_002702167	F: TGATCATCCGGAAGCCTGTG<br>R: ATTGGCAGTACCCTTTCGCT	113	60	98%	0.999
CCL5	NM_175827	F:CACCCACGTCCAGGAGTATT<br>G:AGCGTTGATGTACTCTCGCA	130	60	94%	0.999
Gclm	NM_001038143	F: AGTGGGCACAGGTAAAACCA<br>R: TGCAGTCAAATCAGGTGGCA	77	50	109%	0.993
GSR	NM_001114190	F: GAGATCTCGTACGACTACCTGGTG<br>R: TGGGTACACATCCGACATTCA	145	60	130%	0.960
IL-6	NM_173923	F: ACGAAAGAGAGCTCCATCTGC<br>R: AATGGAGTGAAGGCGCTTGT	71	60	89%	0.992
IL-8	NM_173925	F: TCCAAGCTGGCTGTTGCTC<br>R: TGGGGTGGAAAGGTGTGGAAT	133	60	91%	1.000
IL-1β	NM_853484	F: GCAAGGAGAGGAAAGAGACAACA<br>R: GGGTGGGCGTATCACCTTT	98	60	95%	0.999
IL-20RA	NM_001206342	F:TGGGCAGAAACTGGACGATT<br>R:TCTTTGGAGCCGTCAGAACA	112	60	92%	0.991
Nqo-1	NM_001034535	F: TGGCCAATTCAGAGTGGCA<br>R: GGGAGTGTGCCCAATGCTAT	84	60	127%	0.981
Nrf2	NM_001011678	F:GCATGATGGACTTGGAGCTG<br>R:GCTCATGCTCCTTCTGTCGT	144	60	118%	0.992

Sod1	NM_174615	F: TTCTCTACTTGGTTGGGGCG<br>R: ACGACTGTATCTCCCTTTGCC	145	60	91%	0.998
Sod2	NM_201527	F: GGATCCCCTGCAAGGAACAA<br>R: TGGCCTTCAGATAATCGGGC	110	60	103%	0.999
