# Supplementary material for: Escherichia coli and Staphylococcus aureus Differentially Regulate Nrf2 Pathway in Bovine Mammary Epithelial Cells: Relation to Distinct Innate Immune Response
Source: Cells. 2021 Dec 6;10(12):3426. doi: 10.3390/cells10123426 (PMC8700232; doi:10.3390/cells10123426)
Supplement: Supplementary file 1 [file cells-10-03426-s001.zip › cells-1449855-supplementary/supplementary files/Table S2.pdf]

Table S2. Dysregulated genes in response to *S. aureus* stimulation compared to unstimulated control cells

Gene symbol	Gene name	log2FC	FDR	Regulated	Function
**HLA-DRB3**	major histocompatibility complex, class II, DR beta 3 precursor	0.62	3.38E-2	up	peptide antigen binding and MHC class II receptor activity
**Grem1**	gremlin-1 isoform X1	0.65	1.92E-4	up	cytokine activity and BMP binding
**FGFBP1**	fibroblast growth factor-binding protein 1 precursor	0.46	4.14E-3	up	heparin binding and fibroblast growth factor binding
**DDIT4**	DNA damage-inducible transcript 4 protein	0.38	4.26E-2	up	responses to hypoxia and DNA damage
**MYOZ3**	myozenin-3 isoform	-1.21	2.17E-2	down	calcineurin-interacting proteins
**DAPP1**	dual adapter for phosphotyrosine and 3-phosphotyrosine and 3-phosphoinositide	-1.18	2.17E-2	down	phospholipid binding and phosphatidylinositol-3,4-bisphosphate binding
**EGR1**	early growth response protein 1	-0.83	2.87E-4	down	transcriptional regulator
**NF2L3**	nuclear factor erythroid 2-related factor 3	-0.80	1.13E-2	down	DNA-binding transcription factor activity and transcription coactivator activity
**Ckb**	creatine kinase B-type	-0.76	4.14E-6	down	transferase activity, transferring phosphorus-containing groups and kinase activity
**C-fos**	proto-oncogene c-Fos	-0.76	3.54E-6	down	regulators of cell proliferation, differentiation, and transformation
**GADD45B**	growth arrest and DNA damage-inducible protein GADD45 beta	-0.52	2.21E-3	down	regulation of growth and apoptosis
