# Supplementary material for: Escherichia coli and Staphylococcus aureus Differentially Regulate Nrf2 Pathway in Bovine Mammary Epithelial Cells: Relation to Distinct Innate Immune Response
Source: Cells. 2021 Dec 6;10(12):3426. doi: 10.3390/cells10123426 (PMC8700232; doi:10.3390/cells10123426)
Supplement: Supplementary file 1 [file cells-10-03426-s001.zip › cells-1449855-supplementary/supplementary files/Table S3.pdf]

Table S3. Dysregulated Nrf2 downstream target genes in response to *E. coli* and *S. aureus* stimulation compared to unstimulated control cells.

General biochemical function	Symbol	Name	*E. coli*			*S. aureus*
			log2FC	FDR	regulated	regulated
Detoxication: Phase I drug, oxidation, reduction and hydrolysis	ABCC4	ATP-binding cassette subfamily C member 4, also known as multidrug resistance-associated protein 4	0.61	3.67E-06	up	normal
	ALDH1A3	aldehyde dehydrogenase 1 family, member A3	0.74	1.02E-03	up	normal
	ALDH7A1	aldehyde dehydrogenase 7 family, member A1	0.28	1.57E-02	up	normal
Drug transporter	CYP3A4	cytochrome P450, family 3, subfamily A	1.43	5.08E-46	up	normal
	EPHX2	epoxide hydrolase 2	1.32	9.04E-04	up	normal
	GSR1	glutathione reductase	0.46	8.97E-04	up	normal
Antioxidant: GSH-based system	GPX8	glutathione peroxidase 8	0.44	1.48E-02	up	normal
	SLC6A9	glycine transporter	3.96	7.28E-46	up	normal
	GST	Glutathione S-transferase	0.61	1.94E-02	up	normal
	SLC1A1	Solute carrier family 1 (neuronal/epithelial high affinity glutamate transporter, system Xag), member 1	0.54	6.24E-07	up	normal
Antioxidant: TXN-based system	TXNRD1	thioredoxin reductase 1	1.85	1.70E-29	up	normal
	Trx	thioredoxin	0.33	2.72E-03	up	normal
	TXNIP	thioredoxin-interacting protein	0.91	2.71E-03	up	normal
Antioxidant enzyme	SOD2	superoxide dismutase 2	1.85	1.70E-29	up	normal
Carbohydrate metabolism and NADPH regeneration	ME1	malic enzyme 1, NADP+-dependent, cytosolic	0.63	3.10E-11	up	normal
Lipid metabolism: lipases	LIPH	lipase, member H	0.44	3.76E-05	up	normal
